# Correlation analysis of Serum Hcy, HMGB1, and TLR4 levels and mononuclear macrophage polarisation in acute cerebral infarction

**DOI:** 10.5937/jomb0-61705

**Published:** 2026-02-27

**Authors:** Han Gao, Yin-Xin, Tao-Tao Tan, Jin Hu

**Affiliations:** 1 Affiliated Hospital of Jiaxing University, Department of Neurology, Jiaxing City, China; 2 Xiangya Hospital of Central South University, Laboratory Department, Changsha City, China

**Keywords:** acute cerebral infarction, homocysteine, high mobility group protein B1, toll-like receptor 4, mononuclear macrophage polarisation, prognosis-related analysis, akutni cerebralni infarkt, homocistein, protein B1 visoke pokretljivosti, "Toll-like" receptor 4, polarizacija mononuklearnih makrofaga, analiza povezana sa prognozom

## Abstract

**Background:**

To explore the correlations between homocysteine (Hcy), high mobility group protein B1 (HMGB1), and Toll-like receptor 4 (TLR4) levels and the polarisation of mononuclear macrophages among individuals who were admitted with acute cerebral infarction (ACI).

**Methods:**

The case group comprised 214 ACI patients admitted to the hospital between August 2022 and August 2024, whereas the control group comprised 100 healthy individuals who underwent physical examinations during the same period. A comparison was made between the two groups' admission serum Hcy, HMGB1, and TLR4 levels. The polarisation conditions of the mononuclear macrophages in the two groups were compared [proportion of M1-type cells, proportion of M2-type cells, M1/M2 ratio, M1-type polarisation markers (interleukin-1b, tumour necrosis factor-a), and M2-type polarisation markers (interleukin-10, transforming growth factor-b)]. The aim was to investigate the connections between the polarisation of mononuclear macrophages and the levels of Hcy, HMGB1, and TLR4 in ACI patients, as well as the associations between these levels and the prognosis of ACI patients. Serum levels of Hcy, HMGB1, and TLR4 were compared between ACI patients grouped by excellent or poor prognosis. The prognostic efficacy of Hcy, HMGB1, and TLR4 in ACI patients was evaluated using receiver operating characteristic (ROC) curves.

**Results:**

Serum Hcy, HMGB1, and TLR4 levels in the case group were considerably higher (P&lt; 0.05) than those in the control group. Interleukin-1p and tumour necrosis factor-a levels, the M1/M2 ratio, and the percentage of M1-type cells were all considerably greater (P&lt; 0.05) in the case group compared to the control group, although transforming growth factor-b and interleukin-10 levels were significantly lower (P&lt; 0.05). Serum Hcy, HMGB1, and TLR4 levels in ACI patients were found to be negatively correlated with interleukin-10 and transforming growth factor-b (P&lt; 0.05) and positively correlated with the proportion of M1-type cells, the M1/M2 ratio, and the levels of interleukin-1b and tumour necrosis factor-a (P&lt; 0.05), according to the results of the Pearson correlation analysis. Serum Hcy, HMGB1, and TLR4 levels were substantially higher (P&lt; 0.05) in the poor prognosis group than in the good prognosis group. With area under the curve (AUC) values of 80.00%, 77.80%, and 0.840 for sensitivity, specificity, and AUC, respectively. The ROC curve analysis showed that the combined prediction of Hcy, HMGB1, and TLR4 levels had a comparatively high efficacy in predicting the prognosis of ACI patients.

**Conclusions:**

Patients with ACI had elevated Hcy levels and increased HMGB1 and TLR4 expression in peripheral blood. These levels are anticipated to serve as biomarkers for the clinical diagnosis and treatment of ACI and are strongly correlated with mononuclear macrophage polarisation and patient prognosis.

## Abstract (sr)

**Uvod:**

Cilj je bio da se istraži korelacija između nivoa homocisteina (Hcy), proteina B1 visoke pokretljivosti u grupi HMG (HMGB1) i "Toll-like" receptora 4 (TLR4) i polarizacije mononuklearnih makrofaga kod pacijenata primljenih sa akutnim cerebralnim infarktom (ACI).

**Metode:**

Grupu ispitanika činilo je 214 pacijenata sa ACI primljenih u bolnicu između avgusta 2022. i avgusta 2024. godine, dok je kontrolnu grupu činilo 100 zdravih osoba koje su prošle sistematski pregled u istom periodu. U poređivani su nivoi Hcy, HMGB1 i TLR4 u serumu pri prijemu između dve grupe. Procenjeno je stanje polarizacije mononuklearnih makrofaga, uključujući udeo M1 tipa celija, udeo M2 tipa celija, odnos M1/M2, markere polarizacije M1 tipa (interleukin-1b, tumor nekrozni faktor-a) i markere polarizacije M2 tipa (interleukin-10, transformišući faktor rasta-b). Analizirane su veze između polarizacije makrofaga i nivoa Hcy, HMGB1 i TLR4. Takođe, pacijenti sa ACI su stratifikovani prema prognozi, a nivoi Hcy, HMGB1 i TLR4 u serumu upoređeni su između pacijenata sa dobrim i lošim ishodom.

**Rezultati:**

Nivoi Hcy, HMGB1 i TLR4 u serumu kod pacijenata sa ACI bili su značajno viši (P&lt; 0,05) u odnosu na kontrolnu grupu. Nivoi interleukina-1b i TNF-a, odnos M1/M2 i udeo M1 tipa celija bili su značajno veći (P&lt; 0,05), dok su nivoi transformišućeg faktora rasta-b i interleukina-10 bili značajno niži (P &lt; 0,05) u poređenju sa kontrolnom grupom. Nivoi Hcy, HMGB1 i TLR4 bili su značajno viši (P&lt; 0,05) kod pacijenata sa lošom prognozom u odnosu na one sa dobrom prognozom. ROC analiza je pokazala da kombinovano predviđanje nivoa Hcy, HMGB1 i TLR4 ima relativno visoku efikasnost u predviđanju prognoze pacijenata sa ACI.

**Zaključak:**

Pacijenti sa ACI imaju povišene nivoe Hcy i povećanu ekspresiju HMGB1 i TLR4 u perifernoj krvi. Ovi nivoi mogu služiti kao biomarkeri za kliničku dijagnozu i lečenje ACI i snažno su povezani sa polarizacijom mononuklearnih makrofaga i prognozom kod pacijenata.

## Introduction

Acute cerebral infarction (ACI) is an ischemic disease that accounts for 60% to 80% of all strokes and has a relatively high rate of disability and mortality [1]. Previous statistical data show that the 3-month mortality rate of ACI is 9.0% to 9.6%, and the 12-month mortality rate is 11.4% to 15.4%. The immune response occurs throughout the entire pathological process of ACI [2][3][4]. Important components of the innate immune system, mononuclear macrophages, significantly impact the course of inflammation. Based on their functional polarisation states, mononuclear macrophages can be divided into two types: M1 proinflammatory (M1) and M2 anti-inflammatory (M2) [5]. Different phenotypes have different effects on the onset, progression and prognosis of ACI. High mobility group protein B1 (HMGB1), an inflammatory mediator and proinflammatory cytokine, participates in immune responses and is closely associated with the pathogenesis of ACI [6][7][8]. Toll-like receptor 4 (TLR4) is a transmembrane receptor that can be expressed in mononuclear macrophages, microglia, etc. TLR4 has a key function in the onset and progression of ACI and can mediate inflammatory damage. Homocysteine (Hcy) is a product of methionine metabolism that can promote the generation of oxygen-free radicals, induce vascular endothelial damage, and be related to atherosclerosis [9][10][11]. Although relationships among Hcy, HMGB1, TLR4, and ACI have been reported, relatively few studies have investigated their relationships with the polarisation of mononuclear macrophages [12][13][14].

To measure the levels of Hcy, HMGB1, and TLR4 and the polarisation of mononuclear macrophages in ACI patients at admission, the correlations between the levels of Hcy, HMGB1, and TLR4 and the polarisation of mononuclear macrophages were analysed, and the clinical significance of Hcy, HMGB1, and TLR4 in the diagnosis and treatment of ACI was understood.

## Materials and methods

### General information

The case group consisted of 214 ACI patients admitted to our hospital between August 2022 and August 2024, whereas the control group comprised 100 healthy individuals examined at our hospital during that period.The aforementioned respondents' details, such as age, sex, smoking history (defined as smoking at least one cigarette per day for a minimum of six months), drinking history (drinking at least once a week for a minimum of six months), history of hypertension, coronary heart disease, and type 2 diabetes, were collected in accordance with the International Guidelines for the Prevention and Treatment of Type 1 Diabetes Mellitus (2020 Edition). The case group consisted of 54 males and 53 females. They ranged in age from 30 to 83 years, with an average age of 64.56±3.16 years. There were 16 patients with a history of smoking. There were 19 patients with a history of alcohol consumption. There was no statistically significant difference in sex, smoking history, drinking history or age.

### Inclusion criteria and exclusion criteria

The inclusion criterion for patients was an ACI diagnosis in accordance with the relevant standards in the »Guidelines for the Diagnosis and Treatment of Acute Ischemic Stroke 2020«. First onset; Age: 30-85 years; No mental illness; There was no recent history of severe head trauma.

Exclusion criteria: incomplete medical records; the presence of benign or malignant tumours; coagulation dysfunction; a history of rheumatic diseases; a history of infection within 90 days before enrollment; liver, kidney, heart or lung dysfunction; a history of head surgery; and pregnancy or lactation.

Our hospital's Medical Ethics Committee approved this study (Approval No. XHCSU-22-045), and each patient's family member signed the informed consent form.

### Determination of serum Hcy, HMGB1, TLR4 and polarization marker levels

Four millilitres of fasting elbow venous blood were collected from the subjects within 3 hours after admission. The serum was separated from the blood by centrifuging it for five minutes at 3,000 rpm. Serum Hcy, HMGB1, and TLR4 levels were assessed using the enzyme-linked immunosorbent test (ELISA). Accordingly, Shanghai Guangrui Biotechnology Co., Ltd., Shanghai Jianglai Biotechnology Co., Ltd., and Shanghai Yuanmu Biotechnology Co., Ltd. supplied the kits that were utilised. The levels of M1-type polarisation markers [Interleukin (IL)-1β and tumour necrosis factor-α (TNF-α)] and M2-type polarisation markers were measured by radioimmunoassay. In accordance with the reagent instructions, reagent kits were acquired from Shanghai Kemin Biotechnology Co., Ltd., Shanghai Guduo Biotechnology Co., Ltd., Shanghai Qiaoyu Biotechnology Co., Ltd., and Hangzhou Lianke Biotechnology Co., Ltd.

### Detection of the polarisation of M1 and M2

The M1 markers CCR2 and CD68 were stained with PE-conjugated mouse anti-human chemokine-C receptor-2 (CCR2) and APC-conjugated mouse anti-human leukocyte differentiation antigen 68 (CD68) provided by BD Company of the United States. The M2 markers CX3CR1 and CD163 were stained with FITC-conjugated mouse anti-human chemokine X3C receptor-1 (CX3CR1) and PE-conjugated mouse anti-human leukocyte differentiation antigen 163 (CD163) provided by Aimeijie Technology Co., Ltd. An Invitrogen Attune NxT flow cytometer (Thermo Fisher Scientific) was used to measure the antibody levels on the cell surface. The M1/M2 ratio and the percentages of M1- and M2-type cells were then computed.

### Prognosis judgment

Intravenous thrombolytic therapy was administered within 4.5 hours of patient onset. Alteplase was used for intravenous thrombolysis (50 mg per vial, Boehringer Ingelheim Pharmaceutical Co., Ltd., National Drug Approval No. S20110052) at a dose of 0.9 mg/kg. Ten per cent of the drug was intravenously injected within 60 seconds, and the remaining drug was continuously pumped in within 60 minutes. After intravenous thrombolytic therapy, antiplatelet therapy, brain cell protection, lipid regulation, and symptomatic supportive treatment should be implemented as appropriate. Three months after intravenous thrombolysis, the patients' prognosis was assessed using the modified Rankin Scale (mRS) score. Patients with mRS scores of 0-1 and >1-6 were included.

### Statistical analysis

To perform the statistical analysis, SPSS 22.0 was used. The data, such as Hcy, HMGB1, and TLR4 levels, were normally distributed and are expressed as x̄±s. Count data, such as sex and history of smoking and drinking, were described as the number of cases or percentages. Correlation analysis was conducted via Pearson correlation. The predictive value of each indication was evaluated using receiver operating characteristic (ROC) curves. P values were considered statistically significant if they were less than 0.05.

## Results

### Comparison of the serum Hcy, HMGB1 and TLR4 levels between the two groups

Serum Hcy, HMGB1, and TLR4 levels in the case group were noticeably higher than those in the control group (P<0.05).

The admission serum indicators of patients with acute cerebral infarction admitted during the same period (case group, n = 214) and healthy subjects undergoing physical examination (control group, n = 100) were compared. The results showed that the levels of homocysteine (Hcy), high-mobility group box 1 (HMGB1), and Toll-like receptor 4 (TLR4) in the case group were significantly higher than those in the control group, with differences that were statistically significant (P<0.05). The three indicators showed a consistent increase in the case group, indicating that the body's inflammatory and innate immune-related signals were significantly activated under the ACI state. It provides a solid basis for the subsequent exploration of its correlation with monocyte/macro-phage polarisation and its role in prognosis assessment (Table 1).

**Table 1:** Comparison of serum Hcy, HMGB1, and TLR4 levels between two groups (x̄±s).

Group	n	Hey <br>(μmol/L)	HMGB1 <br>(μg/mL)	TLR4 <br>(ng/mL)
Control <br>group	100	14.25±1.31	1.51±0.37	1.03±0.25
Case <br>group	214	36.79±3.80	9.62±2.25	5.18±1.23
t		54.784	36.250	33.453
P		<0.001	<0.001	<0.001

### Comparison of the polarisation of mononuclear macrophages in the two groups

The percentage of M1-type cells, the M1/M2 ratio, and the levels of TNF-α and IL-1β were significantly higher (P<0.05) in the case group than in the control group, while the levels of TGF-β and IL-10 were significantly lower (P<0.05). See Table 2.

**Table 2:** Comparison of polarisation of two groups of mononuclear macrophages (x̄±s).

Group	n	Proportion of<br>M1 type cells<br>(%)	Proportion of<br>M2 type cells<br>(%)	M1/M2 <br>ratio	M1 polarisation marker		M2 polarisation marker	
					IL-1β <br>(pg/mL)	TNF-α <br>(ng/mL)	IL-10 <br>(pg/mL)	TGF-β <br>(pg/mL)
Control group	100	1.82±0.45	16.59±3.01	0.11±0.02	3.67±0.72	1.28±0.24	12.22±3.02	22.47±5.41
Case group	214	4.13±0.97	15.76±2.84	0.26±0.05	125.70±20.27	3.25±0.80	3.48±0.80	15.06±3.16
t		20.383	1.673	26.783	62.189	23.325	20.136	8.994
P		<0.001	0.096	<0.001	<0.001	<0.001	<0.001	<0.001

The comparison of the polarisation status of mononuclear macrophages in the two groups showed that the proportion of M1-type cells in the case group was significantly increased, the proportion of M2-type cells was decreased, and the M1/M2 ratio was considerably higher than that in the control group (all P<0.05). Consistent with the changes in cell proportion, the levels of M1-type polarisation markers interleukin-1β and tumour necrosis factor-α in the case group were significantly higher than those in the control group, while the levels of M2-type polarisation markers interleukin-10 and transforming growth factor-β were decreased considerably (all P<0.05). The above results indicate that the peripheral immune microenvironment of patients with acute cerebral infarction shows a proinflammatory M1 bias, with the M2 phenotype, which promotes anti-inflammation and tissue repair, suppressed. This suggests that innate immune activation and inflammatory imbalance are involved in the early pathological process of the disease, and provides a biological basis for subsequent exploration of their correlation with Hcy, HMGB1, and TLR4 levels and their impact on prognosis.

### Relationships between Hcy, HMGB1 and TLR4 levels in ACI patients and the polarisation of mononuclear macrophages

The results of the Pearson correlation analysis revealed that the levels of serum Hcy, HMGB1, and TLR4 in ACI patients were positively correlated with the proportion of M1-type cells, the M1/M2 ratio, the level of IL-1β, and the level of TNF-α (P<0.05) and negatively correlated with the levels of IL-10 and TGF-β (P<0.05).

Pearson correlation analysis showed that in ACI patients, the levels of serum Hcy, HMGB1, and TLR4 were all closely related to the M1 orientation of mononuclear macrophages: positively correlated with the proportion of M1 type, the M1/M2 ratio, and IL-1β and TNF-α, and negatively correlated with M2 type markers IL-10 and TGF-β (all P<0.05). It is suggested that the increase of the molecules mentioned above accompanied by enhanced proinflammatory polarisation and suppressed anti-inflammatory repair phenotypes, may be involved in disease progression through innate immunity and risk signalling pathways (Table 3).

**Table 3:** Correlation analysis.

Item	Hcy		HMGBl		TLR4	
	r	P	r	P	r	P
Proportion of<br>M1-type cells	0.490	<0.001	0.426	<0.001	0.471	<0.001
M1/M2 ratio	0.443	<0.001	0.357	<0.001	0.443	<0.001
II-1β	0.457	<0.001	0.405	<0.001	0.435	<0.001
TNF-α	0.397	<0.001	0.377	<0.001	0.451	<0.001
IL-10	-0.442	<0.001	-0.364	<0.001	-0.427	<0.001
TGF-β	-0.383	<0.001	-0.372	<0.001	-0.446	<0.001

### Relationships between the levels of Hcy, HMGB1 and TLR4 and prognosis in patients with ACI

Among the 214 ACI patients, 54 had a poor prognosis and 160 had a good prognosis, for a poor-prognosis rate of 25.23%. Compared with those in the good-prognosis group, Serum Hcy, HMGB1, and TLR4 levels were considerably higher (P<0.05) in the bad-prognosis group. With areas under the curve (AUCs) of 0.840, sensitivities of 77.80%, and specificities of 80.00%, the ROC curve analysis showed that the combined prediction of Hcy, HMGB1, and TLR4 levels had a comparatively high efficacy in predicting the prognosis of ACI patients.

The comparison of the three-month prognosis groups showed that serum Hcy, HMGB1, and TLR4 levels in the poor-prognosis group were significantly higher than in the good-prognosis group (P<0.05), suggesting that these molecules were positively correlated with adverse outcomes. An increase in their levels indicated an increased risk of poor prognosis. ROC analysis revealed that the combined detection of the three had high efficacy in predicting prognosis, with an AUC of 0.840, a sensitivity of 80.00%, and a specificity of 77.80%, superior to the individual indicators. This suggests that combined detection can enhance early identification and risk stratification for patients with poor prognosis and has substantial clinical application value (Table 4 and Table 5).

**Table 4:** Comparison of serum Hcy, HMGB1, and TLR4 levels between the good prognosis group and the poor prognosis group (x̄±s).

Group	n	Hcy (μmol/L)	HMGBl (pg/mL)	TLR4 (ng/mL)
Good prognosis group	160	34.81±2.78	7.07±1.96	4.29±0.92
Poor prognosis group	54	39.96±4.19	10.94±2.57	6.23±1.31
t		5.959	7.153	7.124
P		<0.001	<0.001	<0.001

**Table 5:** Comparison of the efficacy of Hcy, HMGB1, TLR4 alone and in combination for predicting the prognosis of ACI patients.

Item	Sensitivity (%)	Specificity (%)	Accuracy (%)	Best Truncation Value	AUC (95%CI)
Hcy	55.60	85.02	70.31	37.68 μmol/L	0.721 (0.597~0.843)
HMGB1	74.11	85.01	72.22	9.10 μg/mL	0.746 (0.638~0.851)
TLR4	74.11	78.70	76.41	5.17 ng/mL	0.778 (0.667~0.888)
Hcy+HMGB1+TLR4	77.80	80.00	78.90	-	0.840 (0.751~0.929)

## Discussion

Intravenous thrombolytic therapy for ACI patients to restore cerebral perfusion and preserve the ischemic penumbra is currently recognised as an effective treatment [15]. However, owing to the 4.5-hour time window and the limited rate of vascular dilation, only some patients benefit. Even if they survive, they are accompanied by varying degrees of functional impairment. Therefore, this study aimed to explore potential intervention targets from the perspective of ACI pathophysiology [16][17][18].

Ongoing research has confirmed that the immune system may play a role in the pathophysiology of ACI, and stroke immunity has gradually become a clinical research hotspot [19]. Mononuclear macrophages are important immune cells in the body that originate from bone marrow hematopoietic progenitor cells. They can regulate the body's immunity and play an anti-infection role. Mononuclear macrophages can be activated by various stimuli and differentiate into M1 or M2 macrophages. This process is called polarisation. In recent years, the polarisation of mononuclear macrophages during the occurrence and development of various diseases has received extensive attention. Characteristics of M1-type cells: After activation, antigen presentation is enhanced, and proinflammatory cytokines such as IL-1β, TNF-α, IL-12, and IL-23 are released in large quantities, and high levels of CD80, CD86, etc. are expressed [20][21][22]. Characteristics of M2-type cells: These cells express large amounts of IL-10, TGF-β, IL-12, CD23, CD163, etc., and can stabilise the immune microenvironment of neurons. At present, there are relatively few experimental studies on mononuclear macrophages in ACI, and most focus on animal experiments [23][24][25]. A previous study [26] revealed that, in the acute phase, mice with middle cerebral artery occlusion exhibit a significant reduction in spleen volume, and the number of mononuclear macrophages within the spleen is also significantly decreased. The phenotype of mononuclear macrophages changed significantly after ACI. From the perspective of ACI immunity, an inflammatory cascade occurs in the acute phase, with increased secretion of proinflammatory cytokines that mediate secondary inflammatory damage [27].

HMGB1 is a nonhistone chromatin-binding protein widely distributed in mammalian cells. Within cells, it can participate in nucleosome construction, regulate gene transcription, and be involved in DNA recombination and repair. HMGB1 is the initiator and promoter of the inflammatory cascade, with vigorous inflammatory activity [28][29]. As demonstrated by the study's findings, HMGB1 is strongly expressed in ACI and has a favourable correlation with the percentage of M1-type cells, the M1/M2 ratio, and the concentration of M1-type polarisation antibodies. Compared with the good-prognosis group, the serum HMGB1 level in the poor-prognosis group was significantly higher [30]. The AUC, sensitivity, and specificity of serum HMGB1 in predicting the prognosis of ACI patients were 0.746, 59.30%, and 85.01%, respectively, suggesting that HMGB1 is closely associated with the occurrence, progression, and outcome of ACI. Some scholars believe that when cerebral ischemia occurs, HMGB1 is released from cells, glial cells are activated to induce neuroinflammation, and HMGB1 is further synthesised, forming a vicious cycle. Another view holds that HMGB1 can cause abnormal changes in DNA configuration; regulate the generation of transcriptional complexes; and further participate in DNA transcription, replication, repair, etc. HMGB1 can also be secreted by necrotic or degenerated cells, inducing chemotactic effects and subsequently participating in the inflammatory process.

TLR4 is an important member of the TLR family [31]. They can participate in regulating the body's immune and inflammatory responses, recognising pathogens, and activating innate immunity. In the nervous system, TLR4 can be expressed on microglial cell surfaces. The TLR4 protein level in astrocytes increases significantly under inflammatory conditions. Some scholars have reported that Tianma Gouteng decoction can improve neurological function in rats with cerebral haemorrhage and alleviate cerebral oedema by reducing TLR4 levels. Hcy is a sulfur-containing amino acid. Under physiological conditions, Hcy is present at relatively low levels [32]. When pathological factors cause metabolic disorders of Hcy, it can lead to Hcy accumulation within cells and its entry into the bloodstream, thereby increasing its level. Hcy can promote vascular inflammation and induce atherosclerosis through multiple mechanisms and pathways. Studies have shown that high serum Hcy levels are a risk factor for atherosclerotic vascular disease and can increase the prevalence of ACI. Through a mechanism that is dependent on reactive oxygen species (ROS), Hcy can activate the NOD-like receptor heat protein domain-associated protein 3 (NLRP3) inflammasome in macrophages [33]. This study revealed that Hcy is highly expressed in the peripheral blood of ACI patients and is related to the polarisation of mononuclear macrophages. The detection of Hcy levels in patients with different prognoses revealed that the poor-prognosis group had a considerably higher Hcy level than the good-prognosis group. The AUC, sensitivity, and specificity of Hcy in predicting the prognosis of ACI patients were 0.721, 55.60%, and 85.02%, respectively, suggesting that it has some predictive value.

## Conclusion

Patients with ACI had elevated Hcy levels and increased HMGB1 and TLR4 expression in peripheral blood. These levels are closely associated with mononuclear macrophage polarisation and patient outcomes after intravenous thrombolysis. They are anticipated to become biomarkers for the clinical diagnosis and management of ACI. This study's sample size is constrained, though. Further research is needed to determine the precise process, and increasing the sample size remains necessary to achieve more accurate results.

## Dodatak

### Funding

This work was funded by the Supporting Discipline of Neurology in Jiaxing (2023-ZC-006), the »Morning Star« project of the Affiliated Hospital of Jiaxing University(2022-QMX-026) and the Clinical research project of the First Hospital of Jiaxing (2024-LC-002).

### Conflict of interest statement

All the authors declare that they have no conflict of interest in this work.
